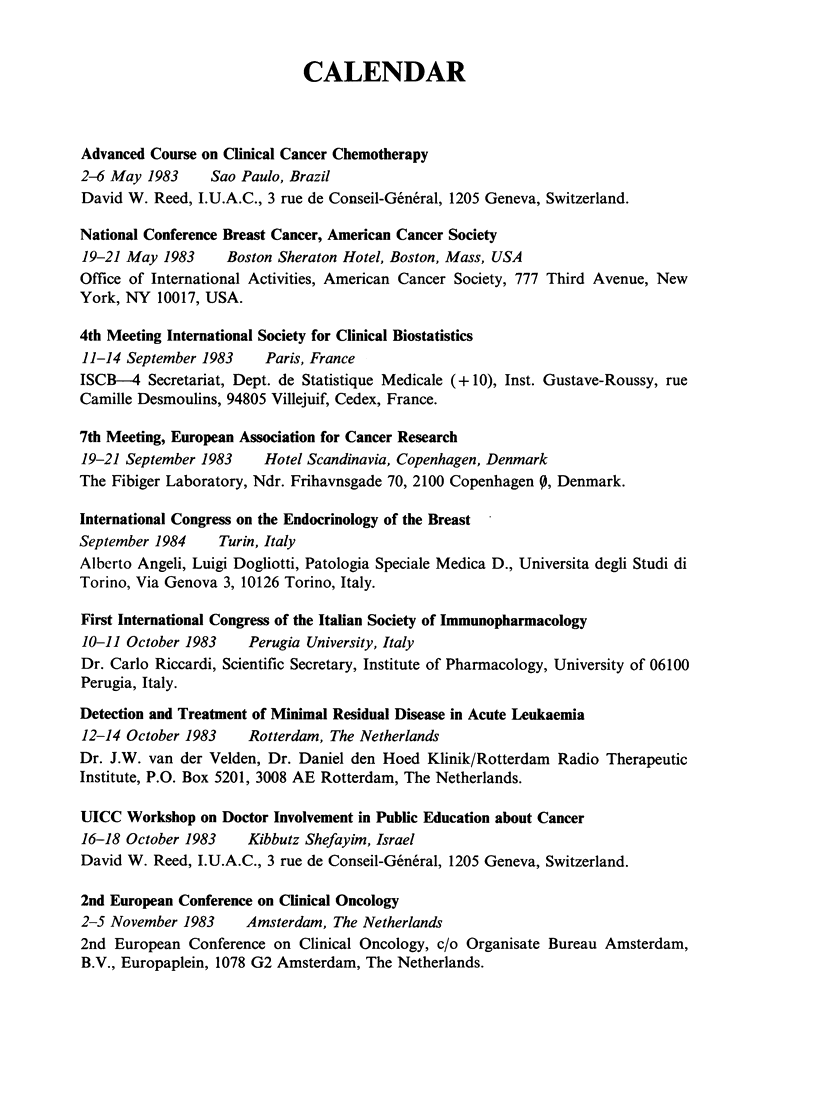# Calendar

**Published:** 1983-04

**Authors:** 


### CALENDAR

**Advanced Course on Clinical Cancer Chemotherapy**
*2–6 May 1983 Sao Paulo, Brazil*
David W. Reed, I.U.A.C., 3 rue de Conseil-Général, 1205 Geneva, Switzerland.

**National Conference Breast Cancer, American Cancer Society**
*19–21 May 1983 Boston Sheraton Hotel, Boston, Mass, USA*
Office of International Activities, American Cancer Society, 777 Third Avenue, New York, NY 10017, USA.

**4th Meeting International Society for Clinical Biostatistics**
*11–14 September 1983 Paris, France*
ISCB—4 Secretariat, Dept. de Statistique Medicale (+10), Inst. Gustave-Roussy, rue Camille Desmoulins, 94805 Villejuif, Cedex, France.

**7th Meeting, European Association for Cancer Research**
*19–21 September 1983 Hotel Scandinavia, Copenhagen, Denmark*
The Fibiger Laboratory, Ndr. Frihavnsgade 70, 2100 Copenhagen Ø, Denmark.

**International Congress on the Endocrinology of the Breast**
*September 1984 Turin, Italy*
Alberto Angeli, Luigi Dogliotti, Patologia Speciale Medica D., Universita degli Studi di Torino, Via Genova 3, 10126 Torino, Italy.

**First International Congress of the Italian Society of Immunopharmacology**
*10–11 October 1983 Perugia University, Italy*
Dr. Carlo Riccardi, Scientific Secretary, Institute of Pharmacology, University of 06100 Perugia, Italy.

**Detection and Treatment of Minimal Residual Disease in Acute Leukaemia**
*12–14 October 1983 Rotterdam, The Netherlands*
Dr. J.W. van der Velden, Dr. Daniel den Hoed Klinik/Rotterdam Radio Therapeutic Institute, P.O. Box 5201, 3008 AE Rotterdam, The Netherlands.

**UICC Workshop on Doctor Involvement in Public Education about Cancer**
*16–18 October 1983 Kibbutz Shefayim, Israel*
David W. Reed, I.U.A.C., 3 rue de Conseil-Général, 1205 Geneva, Switzerland.

**2nd European Conference on Clinical Oncology**
*2–5 November 1983 Amsterdam, The Netherlands*
2nd European Conference on Clinical Oncology, c/o Organisate Bureau Amsterdam, B.V., Europaplein, 1078 G2 Amsterdam, The Netherlands.